# Does the Coronavirus (COVID-19) Pandemic Call for a New Model of Older People Care?

**DOI:** 10.3389/fpubh.2020.00311

**Published:** 2020-06-18

**Authors:** Leonardo Palombi, Giuseppe Liotta, Stefano Orlando, Leonardo Emberti Gialloreti, Maria Cristina Marazzi

**Affiliations:** ^1^Department of Biomedicine and Prevention, University of Tor Vergata, Rome, Italy; ^2^Department of Human Sciences, LUMSA University, Rome, Italy

**Keywords:** novel coronavirus SARS-CoV-2, coronavirus disease 2019, COVID-19, Italy, older people, frailty, public health system

## Introduction

With its rapid global spread, the SARS-COV-2 virus has revealed the fragility of many national public health services as well as their inadequate epidemic preparedness (1). This is the case even in countries that usually play leadership roles in global health, such as the United States or the United Kingdom (2). COVID-19 has exposed several unresolved healthcare issues. Insufficient capacity in many intensive care units soon became a pressing topic that has triggered public alarm in several western countries, particularly in Italy, one of the most gravely affected countries (3, 4). However, it should not be forgotten that this is mainly a public health crisis in relation to both the territorial and residential categories of health settings (5).

## COVID-19 in Italy and The Older People

An important and well-known pandemic driver has been the spread of infection among front-line health personnel caused by the dearth of protective equipment and inadequate training (6, 7). Another driver has been rapid spreading within closed communities, such as nursing homes (3). In Italy, COVID-19 has seriously affected long-term health facilities for the older people and the disabled, nursing homes, and rehabilitation clinics. The Italian National Institute of Health published data from a survey conducted with a sample of 577 residential facilities for the older people that are 12% of the total number of facilities currently operating in the country. Between February 1 and April 6, there were 3,859 deaths (8.4% of total guests). Approximately 50% of the deceased COVID-19 patients resided in Lombardia, the most seriously affected Italian region. According to a research by the Istituto Superiore di Sanità (ISS), 85.9% of the facilities reported a shortage of personal protective equipment for guests and workers, and 17.3% reported cases of infections among their healthcare professionals (8). The susceptibility and vulnerability of these structures are intrinsic: they concentrate many older adults and frail people with high demands for personal care that necessitate close social contact between hosts and personnel. Compounding the issue is that the staff is often unprepared to deal with events of this magnitude (9). Complying with the World Health Organization (WHO) recommendations for measures to adopt in long-term care facilities (LTCF) to combat the COVID-19 epidemic (10) is often impossible in these settings. Several other European and North American countries experienced the same problem, with LTCF heavily hit by the pandemic, resulting in high death tolls (11, 12). In a number of Italian regions, including Lazio, the epidemic is presently contained fairly well within the community; however, it continues to ravage retirement homes and LTCF, thereby confirming that these facilities and their personnel now represent the major epidemic driver (13). Surprisingly, these entities have been often used to host paucisymptomatic COVID-19 patients, thus resulting in further expansion of the epidemic through both new guests and health personnel (14, 15). As a matter of fact, in Italy higher LTCF bed-rates appear to be associated with higher infection-rates among older adults (16).

The devastation caused by the COVID-19 pandemic calls for a new model of care for older adults. Many professionals of the health sector recognize that social interventions at community level and home health care for the frail and older people are effective alternatives in terms of care quality and cost-effectiveness (17, 18). The specific role of residential and community care is different from country to country. However, the COVID-19 crisis seems to recommend a new model of care for older adults, where the balance shifts as much as possible toward community care. The pandemic has shown that, when confronting with these events, community and home care can increase the resilience of health systems much more than residential care. Of course, prioritizing community care is a medium-long term policy, as it needs a wide range of structural changes. Nevertheless, community care services driven by proactive approaches and supported by remote digital teleassistance and communication instruments for real-time monitoring and control of patient conditions may represent a potentially effective alternative to hospital-centered care and residential facilities (19).

To illustrate these concerns, we refer to experiences and results during the COVID-19 pandemic in a social health care program founded in 2004 that originally aimed to lower the mortality and morbidity rates among the older adults caused by unexpected events like heatwaves. The Long Live the Elderly! (LLE) is a community-based pro-active monitoring program (17) based on a practical implementation of social networks that can also be effective during epidemics by counteracting social isolation. LLE is operated by an Italian faith-based organization named Community of Sant'Egidio. It counteracts loneliness and social isolation through continuous efforts to reinforce social relationships in a network of older adults. LLE also facilitates their access to social and health services and monitors persons in need of assistance through a telemedicine program, and it maintains updated health education and training programs. LLE strives to enroll all residents over the age of 80 in activities they organize in wards and neighborhoods in many Italian cities (17, 20, 21).

We compared COVID-19 prevalence and mortality among older people who were included in LLE programs with data related to the over-eighty general populations of Roma and Genova during February and March 2020. These two cities were chosen because updated data on age-specific mortality is available and LLE programs operate in both (22). Beginning on March 8—the official date of the first restrictive decrees for the coronavirus emergency in Italy—the LLE programs enacted emergency protocol similar to their support of participants during heatwaves. The protocol ensures that seniors over the age of 80 are frequently contacted and continuously monitored, and the frailest people have priority. By means of phone calls, the operator assesses each participant's frailty status and emerging needs.

**Figure 1:**
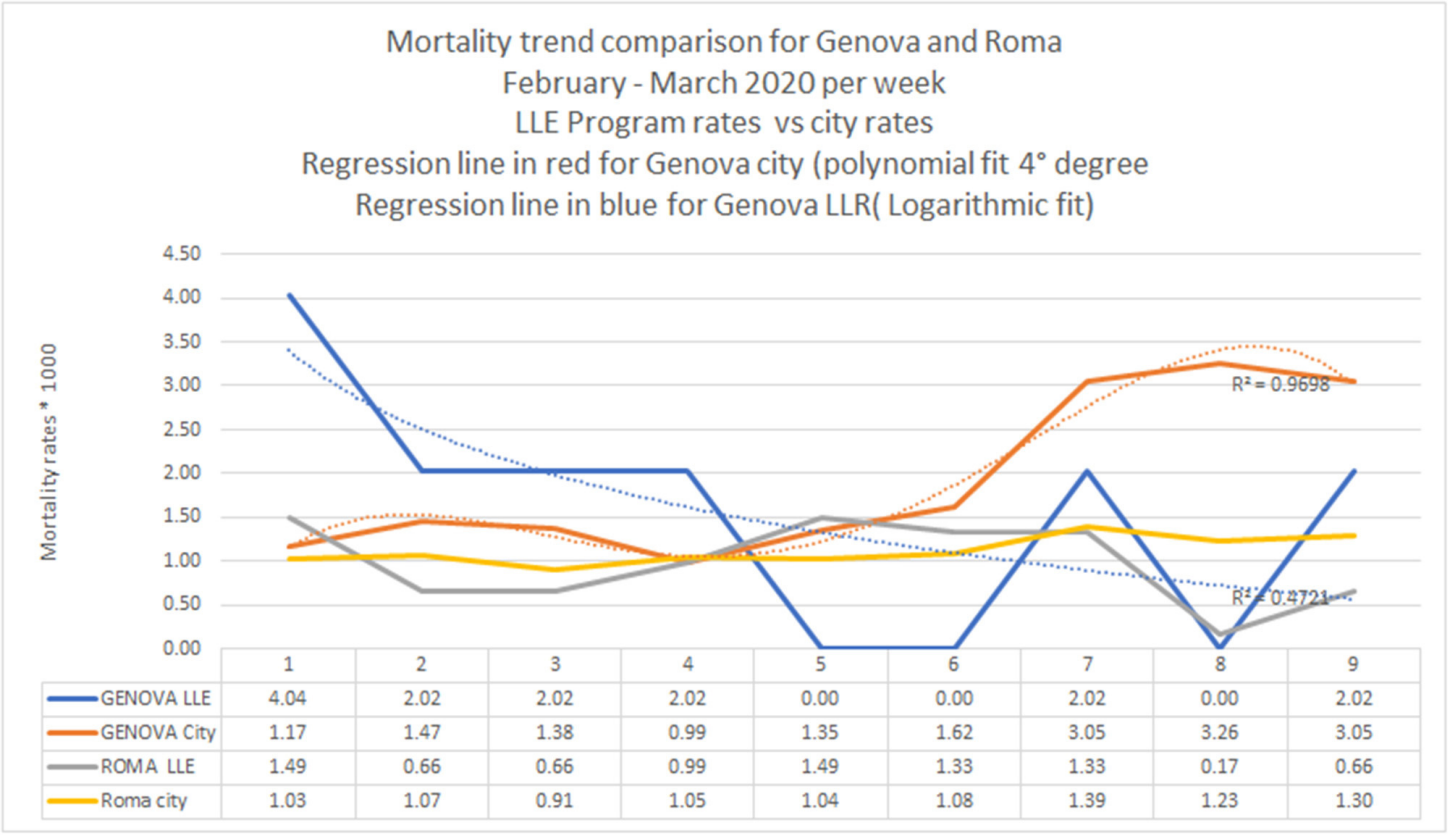
Comparison between the mortality rates of participants in the “Long Live the Elderly!” (LLE) program and the overall mortality rates according to data provided by the Italian health ministry.

From February 1 to March 31, we collected mortality data of people over the age of 80 in the LLE programs of Roma (6,612 persons) and Genova (544 persons) and then compared them with the age-specific mortality rates of the general populations of both cities. Overall, 65% of the monitored older adults were women. From the beginning of March, the vast majority of calls (83%) related to coronavirus emergencies. During these calls, information about government recommendations and measures was provided, current health conditions were monitored, and requests for help were registered. Parallel to these calls, the ordinary activities provided by the program continued (e.g., birthday greetings and regular monitoring calls). In the cities of Roma and Genova, between March 1 and April 14, the following activities were recorded: 15,002 telephone contacts, 1,992 home visits for emergencies and necessary health checks, as well as 314 nutritional support and drug delivery interventions. Figure 1 shows the comparison between the mortality rates of the participants in the LLE program and the overall mortality rates in the two cities included in this survey as provided by the health ministry (22). The differences in the mortality rates trends are clear, particularly in the city of Genova where the overall mortality increased rapidly, while the rate among LLE participants decreased.

Furthermore, among the LLE populations, the indirect standardized death rates were lower than those of the general population in both cities as well as the total population of the two cities. Based on the available age-specific deaths and age-specific death rates calculated for the indirect standardization procedure, the observed and expected deaths among LLE clients were 62 and 83, respectively. The overall standardized death rate was 8.55%0 compared to the 11.64%0 (24) death rate of the general population. We observed a reduction in mortality of more than 25% (SMR = 0.735; 95 CI%: 0.550–0.919). This was consistent with the levels of mitigation achieved by the program during previous serious heatwaves (21), showing the increase in resilience achieved by the program among the older adult population.

## Discussion

The ongoing COVID-19 crisis illustrates the weakness of Italy's residential older people care system, thus evoking proposals for urgent and robust interventions at the community care level through the development of formal and informal support networks. The LLE program is one example of a network that supports the most exposed and frail older adults who keep living at home. Social or more precisely, physical distancing is required. However, this must be accompanied by continuous support in the form of monitoring (e.g., telemedicine), assistance for specific needs (e.g., nutrition and drug supply), disability support, detection of danger signals, timely prevention, and up-to-date information.

Paradoxically, social distancing is only successful in the absence of social isolation, the latter often becoming a survival risk factor in frail populations with high proportions of single residents who are over the age of 80 (23). In order to effectively respect these circumstances, solid social networks should support older adults. In Italy, about one quarter of the population over the age of 75 is socially isolated (23). For most of them, prolonged social distancing is unsustainable, particularly in times such as this when the overcrowding of the health care system has led to reductions or even closures of valuable preventive services. Territorial networks—in addition to their preventive values based on their ability to track and isolate patients in their natural context—also demonstrate a capacity for enhanced resilience that is impossible to achieve in residential facilities and hospitals. We cannot afford to face the next epidemic with an outdated care model. The COVID-19 crisis can open new opportunities for reflection and learning.

## Author Contributions

LP and MM conceived the manuscript. LP, GL, and MM collected the field information. LP, SO, LE, and MM summarized the field information and compared it with published data. LP, GL, and LE performed data analysis. LP, SO, and LE wrote the main text. All authors reviewed, provided input, suggested adjustments to the manuscript drafts, and approved the final version of the text.

## Conflict of Interest

The authors declare that the research was conducted in the absence of any commercial or financial relationships that could be construed as a potential conflict of interest.
